# Associations of gestational thyrotropin levels with disease progression among pregnant women with differentiated thyroid cancer: a retrospective cohort study

**DOI:** 10.3389/fendo.2024.1369344

**Published:** 2024-10-18

**Authors:** Xin Li, Peng Fu, Wu-Cai Xiao, Fang Mei, Fan Zhang, Shanghang Zhang, Jing Chen, Rui Shan, Bang-Kai Sun, Shi-Bing Song, Chun-Hui Yuan, Zheng Liu

**Affiliations:** ^1^ Department of General Surgery, Peking University Third Hospital, Beijing, China; ^2^ Department of Ultrasound, Peking University Third Hospital, Beijing, China; ^3^ Department of Maternal and Child Health, School of Public Health, Peking University, Beijing, China; ^4^ Department of Pathology, Peking University Third Hospital, School of Basic Medical Sciences, Peking University Health Science Center, Beijing, China; ^5^ National Key Laboratory for Multimedia Information Processing, School of Computer Science, Peking University, Beijing, China; ^6^ Information Management and Big Data Center, Peking University Third Hospital, Beijing, China

**Keywords:** differentiated thyroid cancer, thyrotropin, disease progression, pregnancy, DTC

## Abstract

**Purpose:**

Pregnant women with a diagnosis of differentiated thyroid cancer (DTC) were potentially high-risk but largely ignored study population. We aimed to explore whether gestational thyrotropin levels were associated with progression of DTC.

**Methods:**

We conducted a retrospective cohort study at Peking University Third Hospital in Beijing, China from January 2012 to December 2022. We included pregnant women with a pre-pregnancy DTC managed by active surveillance (under-surveillance DTC) or surgical treatment (after-surgery DTC). Dynamic changes of gestational thyrotropin levels across multiple time points were characterized by both statistical (average level, change instability, longitudinal trajectory) and clinical (thyroid dysfunction, thyrotropin suppression, and achievement of thyrotropin suppression target) indicators. Outcomes were clinician-validated progression of DTC, measured separately for patients under surveillance (tumor enlargement or lymph node metastasis) and those after surgery (≥ 3 mm growth in the size of existing metastatic foci, development of new lymph node metastases, ≥ 2 mm growth in the size of existing cancer foci in the contralateral thyroid, or biochemical progression).

**Results:**

Among 43 and 118 patients with under-surveillance and after-surgery DTC, we observed no evidence of associations between any of the quantitative or clinical indicators of gestational thyrotropin levels and progression-free survival, after a median of 2.63 (IQR: 0.90-4.73) and 4.22 (2.53-6.02) year follow-up, respectively (all *P* values > 0.05).

**Conclusions:**

Gestational thyrotropin levels appeared to play a minor role in the progression of under-surveillance or after-surgery DTC. Clinicians might focus on the risk of adverse pregnancy outcomes when optimizing thyrotropin levels for pregnant women with a diagnosis of DTC.

## Introduction

Thyroid cancer has been increasingly prevalent worldwide. Females are much more likely to experience thyroid cancer than males (1). The age-standardized incidence of thyroid cancer has elevated from 6.68/10^5^ to 20.20/10^5^ between 2005 and 2015 among females in China (1). Differentiated thyroid cancer (DTC) makes up more than ninety percent of all types of thyroid cancers (2). DTC is commonly diagnosed in women of reproductive age whose prevalence only follows breast cancer (3).

The clinical decisions for pregnant patients with DTC are generally complex. The complexity lies in the double burden of health risks for pregnant patients with DTC: adverse pregnancy outcomes and progression of DTC. Gestational thyrotropin is one important focus of prenatal care due to its critical function in the maintenance of normal pregnancy. To optimize gestational thyrotropin levels for pregnant women with DTC, it is equally important to elucidate their associations with adverse pregnancy outcomes as well as with the progression of DTC. However, the existing studies have focused more on the former than the latter associations (4, 5).

Another challenge is to assess the repeated measures of thyrotropin across the whole gestation in a fine-grained manner. Studies have mostly used the static, single-point measure, but this might refrain us from determining whether the influence of thyrotropin levels on outcomes is transient on only one occasion or accumulated along the temporal dimension. For example, the risk of the increase in thyrotropin levels for disease progression possibly differs between that occurs only in early pregnancy with subsequent disappearance and that sustains across the whole period of gestation. It is thus paramount to adopt accurate and sensitive indicators to reflect the dynamic changes in thyrotropin levels during early, middle, and late pregnancy. Several studies have used novel indicators or statistical methods to capture the average level (6), instability characteristics (7), or longitudinal trajectory of the repeated measures over time (8, 9), but these approaches have not been applied to investigate the present topic until now, to our knowledge.

“Less is more” has been recognized as the primary treatment strategy for the low-risk DTC (10, 11). Correspondingly, active surveillance has been considered as an alternative to immediate surgery for appropriately selected patients with low-risk DTC (12). Active surveillance refers to the close monitoring of cancer progression during a period without receiving immediate surgery. Despite the overall preferable prognosis of DTC, the degree of disease progression under active surveillance (briefly called “under-surveillance” below) varies greatly across individuals. Specifically, whether the level of thyrotropin relates to the progression of under-surveillance DTC has remained unresolved (13). Moreover, the relevant evidence until now has largely accumulated for the non-pregnant population (6, 14, 15). With the increasing number of pregnant women comorbid with under-surveillance DTC, it is urgent to clarify the associations of gestational thyrotropin levels with the progression of under-surveillance DTC. This has also been identified as an important research gap in the 2017 “Guidelines of the American Thyroid Association for the Diagnosis and Management of Thyroid Disease During Pregnancy and the Postpartum” (16).

To fill the research gaps, our study was focused on pregnant patients with under-surveillance or after-surgery DTC, comprehensively measured the characteristics of changes in gestational thyrotropin levels by multiple indicators, and examined their associations with the risk of biomedical or structural progression of DTC. Dynamic monitoring of gestational thyrotropin levels has been highlighted in the 2022 “Guidelines for prevention and management of thyroid diseases during pregnancy and perinatal period” in China. Findings of our study would thus respond in a timely matter to this call through providing more detailed evidence (17).

## Methods

### Study population

This was a retrospective cohort study conducted at Peking University Third Hospital in Beijing, China from January 2012 to December 2022. First, we selected patients if they had at least two thyrotropin measurements during gestation and were pathologically diagnosed with DTC. Then we selected those who were very similar to those in the prospective active surveillance studies as our *study population 1*: pregnant women with under-surveillance DTC. Inclusion criteria: 1) women receiving surgical treatment after the end of pregnancy; 2) with a duration of surveillance of no less than 6 months prior to surgery; and 3) undergoing at least two examinations of neck ultrasonography prior to surgery; Exclusion criteria: patients with lymph node metastasis or extra-thyroid invasion at baseline. For our *study population 2* (pregnant women with after-surgery DTC), the inclusion criteria were: 1) women receiving surgical treatment before pregnancy; and 2) with postoperational and postpartum examinations of neck ultrasonography, or measurements of serum thyroglobulin (Tg) levels and Tg antibodies. Finally, a total of 43 and 118 patients were included as those with under-surveillance DTC and those with after-surgery DTC, respectively. This study was approved by the Medical Research Ethics Committee of Peking University Third Hospital (No. IRB00006761-M2022721).

### Gestational thyrotropin levels

Thyrotropin levels were measured with commercial kits (Siemens Healthcare Diagnostics) using a fully automatic chemiluminescence immunoassay analyzer (ADVIA Centaur XP; Siemens Healthcare Diagnostics). The lowest and highest detection levels of thyrotropin were 0.008 mIU/L and 150.000 mIU/L, respectively.

Based on literature review (6–9), guidelines (17, 18) and our previous work (19, 20), we used both statistical and clinical indicators to comprehensively reflect the characteristics of the dynamic changes in gestational thyrotropin levels. Following the current Guidelines for the diagnosis and management of thyroid nodules and DTC in China (21), no levothyroxine medication was administered to under-surveillance individuals to maintain low thyrotropin levels while this medication was administered to after-surgery individuals to achieve thyrotropin suppression in this study setting. As such, specific indicators of gestational thyrotropin levels differed between our two study populations. For our study population 1 (pregnant women with under-surveillance DTC), the indicators of gestational thyrotropin levels included (1) time-weighted average of multiple measurements of thyrotropin, (2) instability of change in thyrotropin levels across multiple measurements, (3) longitudinal trajectory of thyrotropin levels across multiple measurements, and (4) thyroid dysfunction during gestation; for our study population 2 (pregnant women with after-surgery DTC), we additionally measured the gestational thyrotropin levels using (5) level of thyrotropin suppression and (6) achievement of thyrotropin suppression target based on response to therapy. Detailed definitions, calculation formula, or classification criteria for these indicators are shown in Supplemental **Table 1**.

**Table 1:** Baseline characteristics of patients with under-surveillance DTC.

Characteristics	N = 43
**Age at conception, year**	33.03 ± 3.47
Tumor type, n (%)	
Papillary thyroid carcinoma	37 (86.05)
Follicular thyroid carcinoma	6 (13.95)
Maximal diameter of tumor, cm	
Papillary thyroid carcinoma	0.82 ± 0.56
Follicular thyroid carcinoma	2.38 ± 0.71
Primary tumor status, n (%)	
T1a	29 (67.44)
T1b	10 (23.26)
T2	4 (9.30)
**Hashimoto’s thyroiditis, n (%)**	19 (44.19)

PTC, papillary thyroid cancer; PTMC, papillary thyroid microcarcinoma.

### Disease progression

The progression of under-surveillance DTC was assessed by tumor size enlargement (one greatest increment in one dimension of tumor size of no less than 3 mm); incident lymph node metastasis (LNM) at follow-up observation; progression (either tumor size enlargement or incident LNM).

The progression of after-surgery DTC was assessed in both structural and biochemical types. Structural progression referred to incident LNM, ≥ 3 mm growth in the size of existing metastatic foci (22), or ≥ 2 mm growth in the size of existing cancer foci in the contralateral thyroid by using neck ultrasonography. Biochemical progression referred to a ≥ 20% increase in postoperational serum Tg or Tg antibodies relative to the preoperational level (23). To ensure the accuracy of Tg measurement, we only included the measurement values of serum Tg if the patient’s Tg antibody was negative (24).

Status of disease progression in clinical records were carefully reviewed and checked by two researchers with rich experiences in clinical practice (X. L.) and data preprocessing (W.C.X.).

### Statistical analyses

We first used Schoenfeld residuals to test the proportional-hazards assumption, and then we adopted Cox proportional risk models to analyze the relationship between indicators of gestational thyrotropin levels and progression-free survival. To estimate the adjusted Hazard Ratio (HR) and its 95% confidence interval (CI), the models among patients with under-surveillance DTC were adjusted for tumor type (papillary thyroid carcinoma; follicular thyroid carcinoma), tumor size at baseline, the status of Hashimoto’s thyroiditis (yes; no), and age at conception; the models among patients with after-surgery DTC were adjusted for tumor size at surgery, the status of Hashimoto’s thyroiditis (yes; no), age at surgery, and surgical type (thyroid lobectomy; total thyroidectomy). Statistical analyses were performed using R software version 4.2 and Stata software version 16.0. P values < 0.05 were considered statistically significant.

## Results

### Baseline characteristics of patients with under-surveillance or after-surgery DTC

**Tables 1**, **2** shows the baseline characteristics of patients with under-surveillance DTC (n = 43) and those with after-surgery DTC (n = 118), respectively. More than 85% of the two groups of the study population were diagnosed with low-risk (T_1_N_0_M_0_) papillary thyroid carcinoma. Approximately 44% patients with under-surveillance DTC and 35% of those with after-surgery DTC were comorbid with Hashimoto’s thyroiditis, respectively.

**Table 2:** Baseline characteristics of patients with after-surgery DTC.

Characteristics	N = 118
**Age at surgery, year**	30.66 ± 4.11
Tumor type, n (%)	
Papillary thyroid carcinoma	114 (96.61)
Follicular thyroid carcinoma	4 (3.39)
Maximum diameter of tumor, cm	
Papillary thyroid carcinoma	1.21 ± 0.80
Follicular thyroid carcinoma	2.88 ± 1.84
Primary tumor status, n (%)	
T1a/TX	61 (51.69)
T1b	43 (36.44)
T2	6 (5.08)
T3a	4 (3.39)
T3b	4 (3.39)
Nodal status at diagnosis, n (%)	
N0	92 (77.97)
N1a	20 (16.95)
N1b	6 (5.08)
Metastases at diagnosis	
M0	118 (100.00)
Recurrence risk after treatment, n (%)	
High	4 (3.39)
Intermediate	16 (13.56)
Low	98 (83.05)
**Hashimoto’s thyroiditis, n (%)**	41 (34.75)
**Extra-thyroid invasion, n (%)**	18 (15.25)
Surgical type, n (%)	
Total thyroidectomy	45 (38.14)
Lobectomy	73 (61.86)

### Associations of gestational thyrotropin levels with progression of under-surveillance DTC

For patients with under-surveillance DTC, the median duration of surveillance before pregnancy was 0.53 year (IQR: 0.17-2.19). As shown in **Table 3**, after a median of 2.63 (IQR: 0.90-4.73) year follow-up among 43 patients with under-surveillance DTC, 17 (53.13%) patients in the higher time-weighted average thyrotropin group (n = 32) and 4 (36.36%) of those in the lower time-weighted average thyrotropin group (n = 11) occurred progression of DTC. Results from the Cox proportional risk model indicated that there was no evidence of difference between the two groups in DTC progression-free survival, with adjustment for tumor type, tumor size, the status of Hashimoto’s thyroiditis, and age at conception [hazard ratio (HR): 1.51; 95% confidence interval (CI): 0.45, 5.03; *P* = 0.504]. There was also no evidence of associations between any of the other three indicators of gestational thyrotropin levels (instability of change, longitudinal trajectory, thyroid dysfunction) and progression of under-surveillance DTC.

**Table 3:** Associations of gestational thyrotropin levels with progression of under-surveillance DTC.

Indicators of gestational thyrotropin levels	N	Tumor size enlargement	Incident LNM	Progression	Crude HR of Progression (95% CI)	*P*	Adjusted HR of Progression (95% CI)^$^	*P*
Time-weighted average								
≤ 2.8 (median)	11	4 (36.36)	0 (0.00)	4 (36.36)	Ref		Ref	
> 2.8 (median)	32	14 (43.75)	3 (9.38)	17 (53.13)	1.37 (0.45, 4.15)	0.583	1.51 (0.45, 5.03)	0.504
Instability of change								
≤ 0.21 (median)	28	10 (35.71)	2 (7.14)	12 (42.86)	Ref		Ref	
> 0.21 (median)	15	8 (53.33)	1 (6.67)	9 (60.00)	1.06 (0.42, 2.62)	0.908	1.00 (0.39, 2.56)	0.998
Longitudinal trajectory								
Decreasing	38	17 (44.74)	3 (7.89)	20 (52.63)	Ref		Ref	
Sustained	4	1 (25.00)	0 (0.00)	1 (25.00)	0.89 (0.12, 6.82)	0.914	0.69 (0.07, 6.61)	0.749
Increasing	1	0 (0.00)	0 (0.00)	0 (0.00)				
Thyroid dysfunction^#^								
Normal	26	11 (42.31)	3 (11.54)	14 (53.85)	Ref		Ref	
Hyperthyroidism or subclinical hyperthyroidism	6	2 (33.33)	0 (0.00)	2 (33.33)	0.62 (0.14, 2.76)	0.532	0.75 (0.14, 3.97)	0.737

LNM, Lymph node metastasis; HR, hazard ratio; CI, confidence interval; Ref, reference group. ^#^: Missing values due to the lack of diagnosis of thyroid dysfunction. ^$^: The models among patients with under-surveillance DTC were adjusted for tumor type (papillary thyroid carcinoma; follicular thyroid carcinoma), tumor size at baseline, the status of Hashimoto’s thyroiditis (yes; no), and age at conception.

### Associations of gestational thyrotropin levels with progression of after-surgery DTC

**Figure 1:**
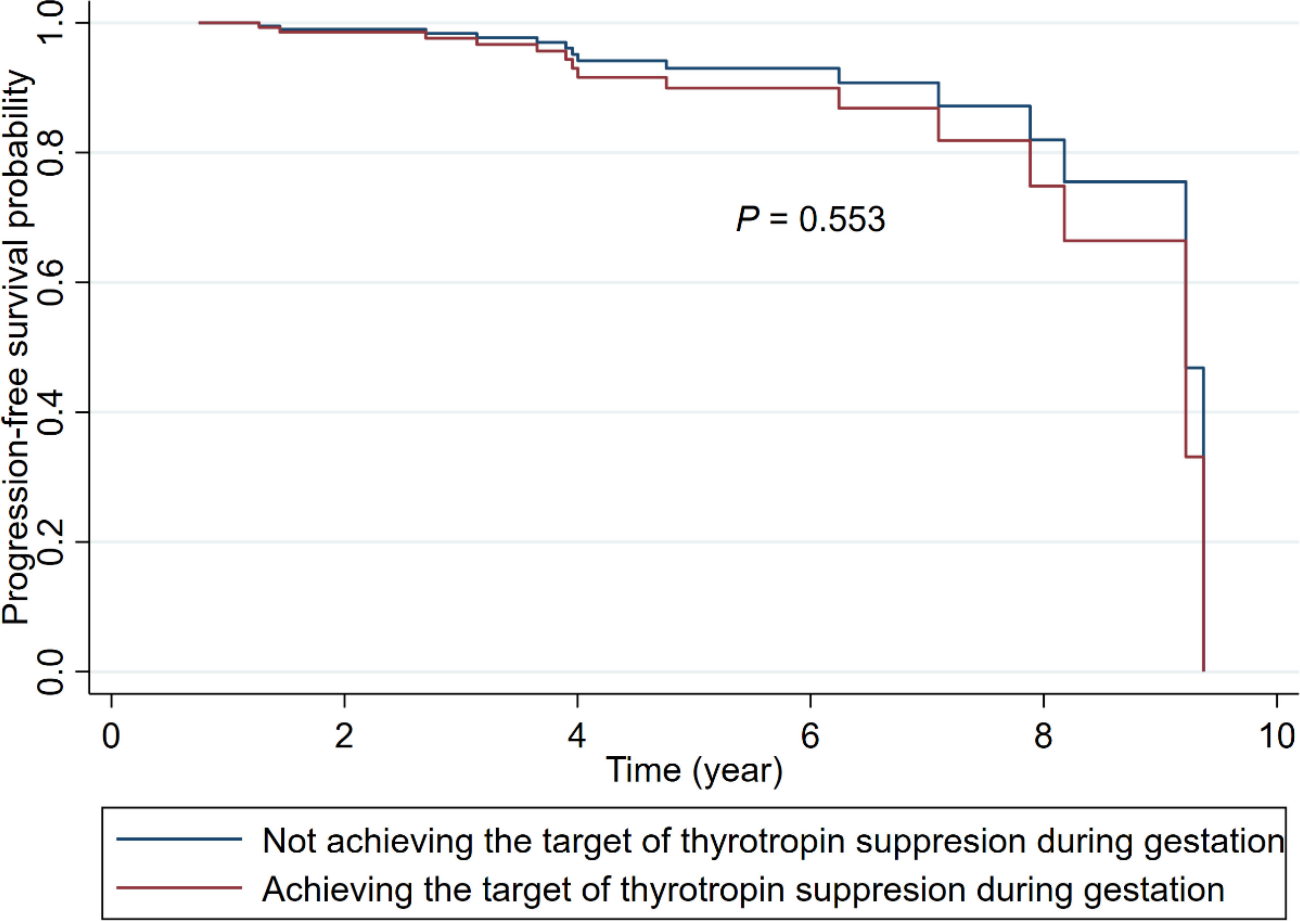
Adjusted Kaplan-Meier survival curves for after-surgery progression of DTC among patients achieving or not achieving the target of thyrotropin suppression during gestation. (The models were adjusted for tumor size at surgery, the status of Hashimoto’s thyroiditis (yes; no), age at surgery, and surgical type (thyroid lobectomy; total thyroidectomy).

For those with after-surgery DTC, the median time interval between surgery and pregnancy was 1.36 year (IQR: 0.62-2.82). As shown in **Table 4**, after a median of 4.22 (IQR: 2.53-6.02) year follow-up among 118 patients with after-surgery DTC, 8 (12.70%) of patients who achieved the target of thyrotropin suppression (n = 63) and 7 (12.73%) of those not achieving the suppression target (n = 55) occurred structural or biochemical progression of DTC, respectively. The Cox proportional risk model results indicated that there was no evidence of difference between the two groups in DTC progression-free survival, with adjustment for tumor size, the status of Hashimoto’s thyroiditis, age at surgery, and surgical type (HR: 1.46; 95% CI: 0.42, 5.05; *P* = 0.553; **Table 4**; **Figure 1**). There was also no evidence of associations between any of the other 4 indicators of gestational thyrotropin levels (time-weighted average, instability of change, longitudinal trajectory, thyroid dysfunction) and the progression of after-surgery DTC (all *P* values > 0.05). Concerning the level of thyrotropin suppression during the gestation, 15 (14.15%) patients occurred after-surgery progression in the no-suppression group (n = 106) while none of the patients showed progression in the mild or complete suppression group (n = 12).

**Table 4:** Associations of gestational thyrotropin levels with progression of after-surgery DTC.

Indicators of gestational thyrotropin levels		N	≥ 2 mm growth in the size of existing cancer foci in the contralateral thyroid	≥ 3 mm growth in the existing metastatic foci	Incident LNM	Biochemical progression	Progression	Crude HR of progression (95% CI)	*P*	Adjusted HR of progression (95% CI)^$^	*P*
Time-weighted average^*^											
≤ 2.8 (median)	56		3 (5.36)	2 (3.57)	2 (3.57)	0 (0.00)	7 (12.50)	Ref		Ref	
> 2.8 (median)	48		2 (4.17)	0 (0.00)	2 (4.17)	2 (4.17)	6 (12.50)	1.03 (0.33, 3.22)	0.956	0.94 (0.25, 3.56)	0.924
Instability of change^*^											
≤ 0.21 (median)	51		4 (7.84)	0 (0.00)	3 (5.88)	1 (1.96)	8 (15.69)	Ref		Ref	
> 0.21 (median)	53		1 (1.89)	2 (3.77)	1 (1.89)	1 (1.89)	5 (9.43)	0.73 (0.23, 2.30)	0.586	0.48 (0.13, 1.80)	0.278
Longitudinal trajectory^*^											
Decreasing	93		5 (5.38)	2 (2.15)	4 (4.30)	1 (1.08)	12 (12.90)	Ref		Ref	
Sustained	5		0 (0.00)	0 (0.00)	0 (0.00)	0 (0.00)	0 (0.00)				
Increasing	6		0 (0.00)	0 (0.00)	0 (0.00)	1 (16.67)	1 (16.67)	1.38 (0.18, 10.80)	0.760	2.78 (0.26, 30.20)	0.401
Thyroid dysfunction											
Normal	25		2 (8.00)	0 (0.00)	1 (4.00)	0 (0.00)	3 (12.00)	Ref		Ref	
Overt or subclinical hyperthyroidism	47		3 (6.38)	1 (2.13)	2 (4.26)	0 (0.00)	6 (12.77)	0.90 (0.22, 3.79)	0.891	1.42 (0.17, 11.77)	0.743
Mixed subclinical hyper- or hypo-thyroidism	3		0 (0.00)	0 (0.00)	0 (0.00)	0 (0.00)	0 (0.00)				
Thyrotropin suppression^#^											
No suppression	106		5 (4.72)	2 (1.89)	4 (3.77)	4 (3.77)	15 (14.15)				
Mild suppression	10		0 (0.00)	0 (0.00)	0 (0.00)	0 (0.00)	0 (0.00)				
Complete suppression	2		0 (0.00)	0 (0.00)	0 (0.00)	0 (0.00)	0 (0.00)				
Thyrotropin suppression achieving target											
No	55		2 (3.64)	1 (1.82)	1 (1.82)	3 (5.45)	7 (12.73)	Ref		Ref	
Yes	63		3 (4.76)	1 (1.59)	3 (4.76)	1 (1.59)	8 (12.70)	1.05 (0.36, 3.04)	0.927	1.46 (0.42, 5.05)	0.553

LNM, Lymph node metastasis; HR, hazard ratio; CI, confidence interval; Ref, reference group. ^*^: Missing values due to the number of measurements of thyrotropin during gestation was less than two. ^#^Missing values due to the lack of diagnosis of thyroid dysfunction. ^$^The models were adjusted for tumor size at surgery, the status of Hashimoto’s thyroiditis (yes; no), age at surgery, and surgical type (thyroid lobectomy; total thyroidectomy).

## Discussion

In this retrospective cohort study, we adopted six distinct indicators to comprehensively characterize gestational thyrotropin levels in terms of the average of thyrotropin level, instability of change in thyrotropin, longitudinal trajectory of thyrotropin, type of gestational thyroid dysfunction, level of thyrotropin suppression, and achievement of thyrotropin target during the pregnancy. We observed no evidence of associations between these indicators of gestational thyrotropin levels and disease progression in both pregnant women with under-surveillance DTC and those with after-surgery DTC.

First, our study highlighted the important clinical question regarding the monitoring and treatment of gestational thyrotropin levels among pregnant women with a DTC history, and answered this question by exploring the associations of gestational thyrotropin levels with the disease progression of DTC. In previous studies, this topic has mainly been explored among the non-pregnant population, with contradictory findings. Among patients under active surveillance, Sugitani et al. (14) did not observe the associations of baseline thyrotropin or mean thyrotropin during follow-up with the progression of T1_a_N_0_M_0_ papillary thyroid microcarcinoma; by contrast, Ito et al. (6) and Kim et al. (15) used time-weighted indicator of thyrotropin levels to account for the unequal time intervals across multiple measurements and found that lower thyrotropin may be related to suppression of enlargement of low-risk papillary thyroid microcarcinoma, especially for patients aged < 40 years. Among patients after surgical treatment, previous studies of associations between thyrotropin levels and progression of DTC have also been focused on the non-pregnant population. Briefly, the effect of thyrotropin suppression in decreasing the risk of recurrence has been generally acknowledged for patients with moderate-to-high-risk DTC but remained inconclusive for patients with low-risk DTC (25–27).

Notably, these findings from the non-pregnant population cannot be directly translated to the pregnant population due to the difference in thyrotropin levels between the pregnancy and non-pregnancy periods. The normal range and change pattern of thyrotropin levels during pregnancy is greatly influenced by the changes in pregnancy-specific hormones such as estrogen and human chorionic gonadotropin from the early, middle, to late pregnancy (28).

Second, our study was advantageous in not only focusing on patients with after-surgery DTC but also on those with under-surveillance DTC. Active surveillance has been acknowledged as an option for appropriately selected low-risk patients with DTC, but follow-up studies among pregnant women have been scarce, especially in China. Our study addressed this gap and the preliminary results did not observe evidence of statistical associations between gestational thyrotropin levels and disease progression in pregnant women with under-surveillance DTC. However, the results were derived from only 43 eligible patients and thus should be validated in future large-sample studies before clinical translation.

Third, our study was novel in capturing the dynamic change of gestational thyrotropin levels by using multiple statistical-method-driven and clinically meaningful indicators. Based on our literature review, previous studies have assessed thyrotropin levels using static, single-point measurement (5, 29, 30), a simple mean of multiple measurements (14), or the time-weighted average of multiple measurements (6, 15). However, these coarsely-grained indicators could only provide an average estimate of thyrotropin levels with little information about the longitudinal trajectory or instability of change over time. To capture the characteristics of multiple measurements of gestational thyrotropin levels in a fine-grained manner, our study took advantage of the latent class trajectory model (8, 9), a statistical method to simultaneously consider repeated measurements as a whole and classify the population into heterogeneous clusters with intra-cluster individuals following similar trajectory of repeated measurements. Additionally, we adopted a formula to calculate the instability of change (i.e., variability of measurements across time points) previously used in other topics (7).

In addition to the statistical-method assessment, our indicators of gestational thyrotropin levels have also considered clinical implications. We carefully evaluated pregnant women’s trimester-specific thyroid dysfunctions, including hyperthyroidism, subclinical hyperthyroidism, hypothyroidism, and subclinical hypothyroidism. We also examined the level of thyrotropin suppression during pregnancy and whether it achieved the target of thyrotropin suppression corresponding to the risk of recurrence. Our findings suggested that approximately half of pregnant patients with after-surgery DTC did not rigorously achieve the target of thyrotropin suppression during pregnancy, but this appeared to have a minor influence on the risk of disease progression.

Findings of our study should be interpreted cautiously. Our results only indicated that thyrotropin levels within the specific gestation period seemed to play a minor role in the risk of disease progression of DTC. This is not a denying to the long-lasting accumulated effect of thyrotropin levels across pre-pregnancy and postnatal periods on the disease progression of DTC. Additionally, the moderate sample size and relatively low incidence of progression limited the statistical power of multiple regression analyses and precluded us from further conducting subgroup analyses. We cannot accurately assess whether maternal advanced age, parity, assisted reproduction, and other factors moderated the exposure-outcome associations. In addition, restricted by the data available, other variables such as information on L-T4 therapy, hCG, and other hormones were not evaluated. These limitations were relatively common in the single institution-based studies. Nevertheless, this type of study had the advantage of accuracy and homogeneity in diagnosis and treatment and high-standard quality control of data integrity. All of the exposures, covariates, and outcomes used in this study had been carefully validated by experienced clinicians, ensuring the reliability of our study findings. Moreover, our study was unique in the focus on pregnant women with DTC, which was a potentially high-risk but largely ignored study population by previous studies.

The findings of our study had important implications for clinical practice. During regular check-ups for maternal thyrotropin levels during pregnancy, clinicians might pay more attention to the clinically meaningful influence on adverse pregnancy outcomes and the concern on disease progression of DTC seemed not much necessary, in most cases, according to findings of our study. Furthermore, clinicians and patients might persist in a long-term observation of thyrotropin levels during the life course, rather than the temporary fluctuations during the gestation period. Considering the severe supply-and-demand imbalance of medical resources, especially for the management of high-risk pregnant women, our findings have significant implications for resource allocation and prioritization in real-world settings.

## Conclusions

Overall, the risk of progression of under-surveillance or after-surgery DTC was not associated with gestational thyrotropin levels. However, the life-long observation of thyrotropin levels might not be omitted for DTC patients. Our findings also reminded clinicians to prioritize the consideration of adverse pregnancy outcomes when optimizing thyrotropin levels for pregnant women previously diagnosed with DTC.

## Data Availability

The raw data supporting the conclusions of this article will be made available by the authors, without undue reservation.
