# Supplementary material for: Associations of gestational thyrotropin levels with disease progression among pregnant women with differentiated thyroid cancer: a retrospective cohort study
Source: Front Endocrinol (Lausanne). 2024 Oct 18;15:1369344. doi: 10.3389/fendo.2024.1369344 (PMC11527675; doi:10.3389/fendo.2024.1369344)
Supplement: Supplementary file 1 [file Table1.docx]

**Associations of gestational thyrotropin levels with disease progression among pregnant women with differentiated thyroid cancer: A retrospective cohort study**

List of supplemental files:

- Supplemental Table 1 Detailed definitions, calculation formular, or classification criteria for the indicators of gestational thyrotropin levels

**Supplemental Table 1** Detailed definitions, calculation formular, or classification criteria for the indicators of gestational thyrotropin levels

| **Indicators of gestational thyrotropin levels** | **Detailed definition, calculation formula, or classification criteria for the indicators** |
| --- | --- |
| Average of multiple measurements of thyrotropin^[1]^ | 1. We first converted the actual TSH values into TSH score to minimize the bias of different measurement values.   - lower than the detection limit→1 score; detectable and < 0.05 mIU/ml → 2 score; - ≥ 0.05 mIU/ml and less than the lower normal limit → 2.5 score; - within the normal range and lower than the mean of the normal ranges → 3 score; - higher than the upper normal limit → 4 score   2. The calculation formula for the average TSH score:  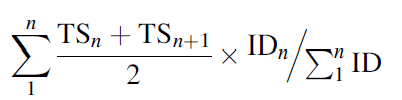  (ID_n_ is each interval (days) and TS_n_ and TS_n+1_ are the TSH scores from the beginning and end of each interval, respectively.) |
| Instability of change in thyrotropin levels across multiple measurements^[2]^ | The calculation formula:  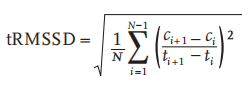  C_i_ (i=1,…, N) are the N measurements of TSH scores and t_i_ (i=1,…,N) are the timestamps at which measurements were recorded, so that t_i+1_–t_i_ is the difference in time between two subsequent measurements. |
| Longitudinal trajectory of thyrotropin levels across multiple measurements | 1. Latent class trajectory modelling (LCTM) is a relatively new methodology in epidemiology to describe life-course exposures, which simplifies heterogeneous populations into homogeneous patterns or classes^[3]^. 2. Taking into account the following trajectory parameters, choose the most suitable number of trajectory classes: 3. APPA (posterior probability of assignments): average of maximum probabilities should be greater than 70% for all classes; 4. OCC (odds of correct classification): OCC values greater than 5.0; 5. Relative entropy: relative entropy values greater than 0.5; 6. Include classes with at least 1% capture of the population |
| Thyroid dysfunction during gestation | We took two steps:  1. Thyroid function for early, middle, and late pregnancy was separately classified as hyperthyroidism, subclinical hyperthyroidism, hypothyroidism, or subclinical hypothyroidism based on the pregnancy-stage-specific measurements of TSH and FT4 levels^[4]^.  2. Thyroid function for each pregnant woman was classified as normal (no records of thyroid dysfunction across the early, middle, and late pregnancy), hyperthyroidism (records of hyperthyroidism or subclinical hyperthyroidism across the early, middle, or late pregnancy), hypothyroidism (records of hypothyroidism or subclinical hypothyroidism during pregnancy), or mixed type (records of both hyperthyroidism/subclinical hyperthyroidism and hypothyroidism/subclinical hypothyroidism during pregnancy). |
| Thyrotropin suppression | Three classifications of thyrotropin suppression^[5]^ including:  1. No suppression, thyrotropin target 0.5 - 2.0 mU/L;  2. Mild suppression, thyrotropin target 0.1 - 0.5 mU/L;  3. Moderate or complete suppression, thyrotropin target < 0.1 mU/L. |
| Achievement of thyrotropin suppression target based on response to thyroid cancer therapy | 1. Patients were classified as achieving thyrotropin targets if their average thyrotropin levels achieved thyrotropin targets corresponding with the response to therapy: no suppression for those with excellent response, mild suppression for those with indeterminate response, and moderate or complete suppression for those with biochemical or structural incomplete response^[5]^.  2. Otherwise, patients were classified as not achieving thyrotropin targets. |

**References:**

1. Ito Y, Miyauchi A, Fujishima M, Noda T, Sano T, Sasaki T, Kishi T, Nakamura T. Thyroid-Stimulating Hormone, Age, and Tumor Size are Risk Factors for Progression During Active Surveillance of Low-Risk Papillary Thyroid Microcarcinoma in Adults. World J Surg. 2023 Feb;47(2):392-401. doi: 10.1007/s00268-022-06770-z.

2. Taquet M, Griffiths K, Palmer EOC, et al. Early trajectory of clinical global impression as a transdiagnostic predictor of psychiatric hospitalisation: a retrospective cohort study. Lancet Psychiatry. 2023;10(5):334-341. doi:10.1016/S2215-0366(23)00066-4.

3. Lennon H, Kelly S, Sperrin M, et al. Framework to construct and interpret latent class trajectory modelling. *BMJ Open*. 2018;8(7):e020683. Published 2018 Jul 7. doi:10.1136/bmjopen-2017-020683.

4. Writing Committee for Guidelines for P, Management o, Thyroid Diseases During P, Perinatal P, Chinese Society of Endocrinology CMA, Women's Health Care Branch of Chinese Preventive Medicine A. Guidelines for prevention and management of thyroid diseases during pregnancy and perinatal period. Chinese Journal of Endocrinology and Metabolism. 2022; 38:539-51.

5. Haugen, B.R., et al., 2015 American Thyroid Association Management Guidelines for Adult Patients with Thyroid Nodules and Differentiated Thyroid Cancer: The American Thyroid Association Guidelines Task Force on Thyroid Nodules and Differentiated Thyroid Cancer. Thyroid, 2016. **26**(1): p. 1-133.
